# Influence of maternal and perinatal complications on therapeutic hypothermia in newborns with low Apgar scores

**DOI:** 10.1590/1806-9282.20231525

**Published:** 2024-07-19

**Authors:** Pedro Teodoro Carlstron, Marina Nóbrega Augusto, Alberto Borges Peixoto, Edward Araujo, Nathalia Mello, Rosiane Mattar, Sue Yazaki Sun

**Affiliations:** 1 Universidade Federal de São Paulo, Paulista School of Medicine, Department of Obstetrics – São Paulo (SP), Brazil.; 2 Universidade de Uberaba, Mário Palmério University Hospital, Service of Gynecology and Obstetrics – Uberaba (MG), Brazil.; 3 Universidade Federal do Triângulo Mineiro, Department of Obstetrics and Gynecology – Uberaba (MG), Brazil.; 4 Amparo Maternal Hospital, Service of Neonatology – São Paulo (SP), Brazil.; 5 Amparo Maternal Hospital, Service of Obstetrics – São Paulo (SP), Brazil.

**Keywords:** Newborn, Apgar score, Therapeutic hypothermia

## Abstract

**OBJECTIVE:**

The aim of this study was to evaluate the impact of therapeutic hypothermia on maternal and perinatal outcomes in newborns with Apgar score<7 at the 5th min.

**METHODS:**

A retrospective cohort study was carried out with 55 newborns who had an Apgar score<7 at the 5th min (35 without and 20 with therapeutic hypothermia) from low-risk pregnancies between 33 and 41 weeks gestation. The Apgar score was calculated through an objective assessment by a neonatologist in the delivery room. Therapeutic hypothermia was indicated by a neonatologist in the delivery room, according to the protocol established by the Brazilian Society of Pediatrics. The maternal and perinatal outcomes of both groups (without and with therapeutic hypothermia) were compared.

**RESULTS:**

A rate of Apgar score<7 at the 5th min was 1.02%. No statistical differences were observed between the two groups (without and with therapeutic hypothermia) regarding maternal/perinatal complications. The presence of maternal/perinatal complications did not increase the odds ratio of neonatal therapeutic hypothermia in newborns with Apgar score<7 at the 5th min.

**CONCLUSION:**

The rate of Apgar score<7 at the 5th min was low, and it was not associated with any maternal/perinatal complications. There was no significant difference in maternal/perinatal complications between newborns who received therapeutic hypothermia and those who did not.

## INTRODUCTION

The Apgar score was developed in 1952 by Dr. Virginia Apgar to evaluate the interference of obstetric conditions with the clinical condition of newborns shortly after delivery. It evaluates five parameters, namely, heart rate, breathing, muscle tone, skin color, and reflexes, in the newborn at the 1st and 5th min, and each of these parameters can be given from 0 to 2 points, where 0 means the parameter was absent and 2 shows the parameter in its best condition. . This score is currently used worldwide to assess the health status of newborns^1^.

The 5th min Apgar score estimates the vitality of the newborn and the effectiveness of neonatal resuscitation, when applied^1^. The highest score is 10, representing the best condition of a newborn, and the lowest scores are generally associated with the findings suggestive of asphyxia in umbilical cord blood. While the Apgar score has some criticisms and considerations, it is very useful in evaluating the success of reanimation procedures^2^.

There are many obstetric, maternal, and neonatal factors, including gestational age, use of anesthetics during delivery, congenital infections, cardiopulmonary alterations in the newborn, prenatal conditions, and birth weight, which can interfere with the Apgar score. An Apgar score that remains low after the 5th min of birth indicates severe asphyxia, so the clinical relevance of the score increases as the scoring decreases and the time of life increases, because low scores after the 5th min are associated with greater morbidity, neurological impairment, and neonatal mortality^3^.

The Apgar score<7 (between 0 and 6) is a warning sign for pediatricians, and such newborns require special attention and care, according to the pathophysiology and the low Apgar grade^4^. Some factors related to neonatal asphyxia are unavoidable, but there are also preventable and reversible causes, as long as there is adequate obstetric care and relevant neonatal care protocols. Therefore, it is important to identify the risk factors for low Apgar scores so that the appropriate approach can be taken, thus reducing perinatal asphyxia^5^.

Therapeutic hypothermia is a controversial treatment used to treat moderate or severe neonatal encephalopathy. While some studies showed a decrease in neonatal deaths, others have reported an increase^6,7^. A low Apgar score was a relevant factor in identifying newborns eligible for therapeutic hypothermia^8^.

This study aims to evaluate the influence of therapeutic hypothermia on maternal and perinatal outcomes in newborns with Apgar score<7 at the 5th min.

## METHODS

A retrospective cohort study was conducted by analyzing the medical records of pregnant women who had their delivery at the Amparo Maternal Hospital, which cares for low-risk obstetric pregnant women in the city of São Paulo between January 2021 and December 2021. This study was approved by the Research Ethics Committee of the Federal University of São Paulo (CAAE: 57974422.7.0000.5505). The consent form was not necessary because it was a retrospective study.

We included singleton pregnancies with newborns who had Apgar scores<7 at the 5th min and excluded cases with incomplete medical records or those that could not be accessed.

The Apgar score is calculated through an objective assessment by a neonatologist in the delivery room. Five parameters are assessed, namely, heart rate, breathing, muscle tone, skin color, and reflexes, with a score between 0 and 2. This assessment takes place between 1st and 5th min of life. If the score at the 5th min is<7, the assessment should be repeated every 5 min until the score is>7 or until the newborn reaches the 20th min of life^1^. The 5th min Apgar score is related to a good predictor of survival in infancy^9^.

Therapeutic hypothermia is indicated by a neonatologist in the delivery room, according to the protocol established by the Brazilian Society of Pediatrics^10^. Evidence of perinatal asphyxia is required, together with signs of hypoxic–ischemic encephalopathy on physical examination. Evidence of perinatal asphyxia can be assessed by cord blood gases with a pH<7.0, by a history of any acute event at the time of delivery that could cause fetal distress, by an Apgar score ≤5 at the 5th min of life, or by the need for positive pressure ventilation for more than 20 min. The presence of hypoxic–ischemic encephalopathy is assessed using criteria defined by Shankaran et al.^11^, before 6 h of life. These criteria consist of assessing the newborn's level of consciousness, spontaneous activity, posture, tone, primitive reflexes, and autonomic system. Alterations in at least three categories are required for the diagnosis of hypoxic–ischemic encephalopathy. Therapeutic hypothermia is contraindicated when the newborn is in situations of pre-death, complex malformations, genetic abnormalities, or when there is already a defined palliative care approach.

The study assessed several variables related to maternal and newborn clinical data, including maternal age, body mass index (BMI), number of pregnancies, number of previous deliveries, gestational age at admission, presence of prematurity, arterial hypertension during pregnancy, gestational diabetes mellitus, fetal growth restriction, positive culture for group B beta-hemolytic *Streptococcus* (GBS), premature rupture of ovular membranes (PPROM), characteristics of amniotic fluid (clear, fluid meconium, thick meconium, blood), labor (induced or spontaneous), type of delivery (vaginal, forceps, cesarean section), birth weight (<2,500 g, 2,500–4,000 g,>4,000 g), neonatal intensive care unit (NICU) admission, neonatal death, length of hospitalization in NCIU, length of invasive ventilation support, use of antibiotics, vasoactive drug, blood transfusions, intracranial hemorrhage, and whether the death was related with neonatal asphyxia. The cases included in the study will be separated into two groups: Group I—newborns without therapeutic hypothermia and Group II—newborns with therapeutic hypothermia.

The GPower 3.1 software (Heinrich-Heine-Universität, Düsseldorf, Germany) was used to calculate the sample size. To assess the association between the presence of neonatal hypothermia and maternal and newborn clinical data, a total sample of at least 52 patients was required. The sample size analysis was based on a w-effect of 0.50, a probability of error α of 0.05, and a power (probability of error 1-β) of 0.80, with five degrees of freedom (Df).

The data were analyzed using SPSS version 20.0 (Chicago, IL, USA) and Prisma GraphPad 7.0 (Boston, MA, USA). Quantitative variables were subjected to the D'Agostino-Pearson normality test and presented as means and standard deviations. Categorical variables were described as absolute and percentage frequencies and presented in tables and graphs. Differences between categorical variables and their proportions were analyzed using the chi-square test. The effect of the groups on the continuous variables was analyzed using the Mann-Whitney test (nonparametric distribution). The odds ratio (OR) for the development of neonatal hypothermia was calculated using binary logistic regression. The significance level adopted for all tests was p<0.05.

## RESULTS

From January 2021 to December 2021, 55 cases with an Apgar score<7.0 at the 5th min were selected at the Amparo Maternal Hospital. The number of deliveries during this period of time was 5,348, corresponding to a rate of 1.02% of low Apgar score at the 5th min. The characteristics of the study population are shown in Table 1. The cases were separated into two groups: Group I—newborns without therapeutic hypothermia (n=35) and Group II—newborns with therapeutic hypothermia (n=20).

**Table 1:** Characteristics of the study population.

		Group I (n=35)	Group II (n=20)	p-value
Maternal age (years)				
	<18	0.0% (0/34)	5.3% (1/19)	0.358§
	18–35	82.4% (28/34)	68.4% (13/19)	0.311§
	>35	17.6% (6/34)	26.3% (5/19)	0.495§
	BMI (kg/m^2^)	26.0 (22.0–34.0)	27.0 (24.0–36.0)	0.096∫
	Number of pregnancies	1 (1–5)	1 (1–5)	0.984∫
Number of previous deliveries				
	Nulliparous	54,3% (19/35)	50.0% (10/20)	0.759§
	Multiparous	45.3% (16/35)	50.0% (10/20)	0.759§
	Gestational age (weeks)	39.0 (33.0–41.0)	40.0 (38.0–41.0)	0.166∫
	Prematurity	11.8% (4/34)	0.0% (0/19)	0.120§
	Arterial hypertension during pregnancy	8.8% (3/34)	5.3% (1/19)	0.638§
	Gestational diabetes mellitus	8.8% (3/34)	5.3% (1/19)	0.638§
	Fetal growth restriction	0.0% (0/35)	0.0% (0/20)	
	Positive culture for GBS	11.8% (4/34)	10.5% (2/19)	0.945§
	PPROM	8.8% (3/34)	22.2% (4/18)	0.178§
Characteristics of amniotic fluid				
	Clear	61.3% (19/31)	75.0% (12/16)	0.517§
	Fluid meconium	9.7% (3/31)	0.0% (0/16)	0.541§
	Thick meconium	25.8% (8/31)	25.0% (4/16)	>0.999§
	Blood	3.2% (1/31)	0.0% (0/16)	>0.999§
Labor				
	Spontaneous	76.5% (26/34)	61.1% (11/18)	0.336§
	Induced	23.5% (8/34)	38.9% (7/19)	0.336§
Type of delivery				
	Vaginal	44.1% (15/34)	52.6% (10/19)	0.579§
	Forceps	0.0% (0/34)	0.0% (0/19)	
	Cesarean section	55.9% (19/34)	47.4% (9/19)	0.579§
Birth weight				
	<2,500 g	23.5% (8/34)	5.3% (1/19)	0.133§
	2,500–4,000 g	70.6% (24/34)	84.2% (16/19)	0.334§
	>4,000 g	5.9% (2/34)	10.5% (2/19)	0.611§
	Apgar score at the 1st min	3.0 (0.0–8.0)	2.0 (0.0–5.0)	0.185∫
	NICU admission	100.0% (35/35)	100% (20/20)	
	NICU length (days)	3.0 (1.0–19.0)	7.0 (1.0–30.0)	0.032∫
	Invasive ventilation support (h)	5.5 (0–456.0)	24.0 (0–480.0)	0.117∫
	Use of antibiotics	30.3% (10/33)	42.1% (8/19)	0.389§
	Vasoactive drug	28.1% (9/32)	57.9% (11/19)	0.035§
	Blood transfusion	18.2% (6/33)	15.8% (3/19)	0.826§
	Intracranial hemorrhage	6.3% (2/32)	26.3% (5/19)	0.044§
	Neonatal death	5.7% (2/35)	5.0% (1/20)	0.911§

Group 1: without hypothermia; Group 2: with hypothermia; BMI: body mass index; GBS: Group B beta-hemolytic ***Streptococcus***; PPROM: premature rupture of ovular membranes; NICU: neonatal intensive care unit; Mann-Whitney

∫ median (minimum–maximum); Chi-square

§ percentage (n/N); p<0.05.

Newborns with therapeutic hypothermia had a longer stay in the NICU (7.0 vs 3.0 days, p=0.032, respectively), a higher prevalence of vasoactive drug (57.9% vs 28.1%, p=0.035) and intracranial hemorrhage (26.3% vs 6.3%, p=0.044), compared to newborns without therapeutic hypothermia. There was no significant differences between the groups in relation to BMI (p=0.096), Apgar score at the 1st min (p=0.185), maternal age<18 years (p=0.358), maternal age between 18 and 35 years (p=0.311), maternal age>35 years (p=0.495), number of pregnancies (p=0.984), nulliparity (p=0.759), multiparity (p=0.759), gestational age at admission (p=0.166), prematurity (p=0.120), arterial hypertension during pregnancy (p=0.638), gestational diabetes mellitus (p=0.638), positive culture for GBS (p=0.945), PPROM (p=0.178), clear amniotic fluid (p=0.517), fluid meconium (p=0.541), thick meconium (p>0.999), blood in the amniotic fluid (p>0.999), spontaneous onset of labor (p=0.336), induction of labor (p=0.336), vaginal delivery (p=0.579), cesarean section (p=0.579), birth weight<2,500 g (p=0.133), birth weight between 2,500 and 4,000 g (p=0.334), birth weight>4,000 g (p=0.611), NICU admission, invasive ventilation support (p=0.117), use of antibiotics (p=0.389), blood transfusion (0.826), and neonatal death (p=0.911). There was no case of neonatal death related to neonatal asphyxia (Table 1).

The vasoactive drug increased the risk of newborn therapeutic hypothermia (OR:3.51, CI 95% 1.07–11.6, p=0.039). The presence of prematurity (p=0.120), arterial hypertension during pregnancy (p=0.638), gestational diabetes mellitus (p=0.638), positive culture for GBS (p=0.945), PPROM (p=0.178), clear amniotic fluid (p=0.517), fluid meconium (p=0.541), thick meconium (p>0.999), blood in the amniotic fluid (p>0. 999), spontaneous onset of labor (p=0.336), induction of labor (p=0.336), vaginal delivery (p=0.579), cesarean section (p=0.579), birth weight<2,500 g (p=0.133), birth weight between 2,500 and 4,000 g (p=0.334), birth weight>4,000 g (p=0.611), NICU length stay (p=0.051), invasive ventilation support (p=0.183), use of antibiotics (p=0.389), blood transfusion (p=0.826), and intracranial hemorrhage (p=0.061) did not increase the OR of neonatal therapeutic hypothermia (Table 2).

**Table 2:** Odds ratio of hypothermia in newborns with Apgar score <7 at the 5th min.

Variable		OR	95%CI	p-value§
Prematurity		0.174	0.009–3.41	0.120
Arterial hypertension during pregnancy		0.574	0.055–5.94	0.638
Gestational diabetes mellitus		0.574	0.055–5.94	0.638
Positive culture for GBS		0.95	0.159–5.01	0.945
PPROM		2.95	0.582–15.0	0.178
Characteristics of amniotic fluid				
	Clear	1.89	0.533–6.26	0.517
	Fluid meconium	0.00	0–2.20	0.541
	Thick meconium	0.95	0.27–3.96	>0.999
	Blood	0.00	0–17.44	>0.999
Beginning of labor				
	Spontaneous	0.48	0.142–1.68	0.336
	Induced	2.06	0.592–7.02	0.336
Type of delivery				
	Vaginal	1.40	0.438–4.11	0.579
	Cesarean section	0.71	0.243–2.28	0.579
Birth weight				
	<2,500 g	0.18	0.015–1.16	0.133
	2,500–4,000 g	2.22	0.568–8.33	0.334
	>4,000 g	1.88	0.272–12.67	0.611
	NICU length	1.09	1.00–1.19	0.051
	Invasive ventilation support	1.00	0.99–1.08	0.183
	Use of antibiotics	1.67	0.51–5.42	0.389
	Vasoactive drug	3.51	1.07–11.6	0.039
	Blood transfusion	0.84	0.18–3.85	0.826
	Intracranial hemorrhage	5.36	0.92–31.08	0.061

Group 1: without hypothermia; Group 2: with hypothermia; CI: confidence interval; GBS: Group B beta-hemolytic *Streptococcus*; PPROM: premature rupture of ovarian membranes; OR: odds ratio; binary logistic regression: percentage (n/N); p<0.05.

§ Chi-square.

## DISCUSSION

A low Apgar score at the 5th min is associated with nonresponse to adequate resuscitation. The Apgar score at the 5th min is a good predictor of neonatal mortality^12^ and severe morbidities such as neurological disabilities (seems to persist many years postnatally)^13^ and pediatric infections^14^. Low Apgar scores at the 5th min have been associated with several maternal/obstetric complications, including non-vertex fetal presentation, prolonged labor, presence of meconium in amniotic fluid, induced labor, and low birth weight^15^. In Southwest China, hypertensive disorders in pregnancy were a more important factor for a low Apgar score (<7)^16^. In a study developed in Atlanta, Georgia, USA, maternal hypertensive disorders, PPROM, cesarean section, vaginal delivery after cesarean section, and male gender were associated with Apgar score<7 at the 5th min^17^. In our study, maternal arterial hypertension disorders, PPROM, thick meconium in amniotic fluid, induced labor, cesarean section, and birth weight<2,500 g were 7.5%, 25.5%, 13.4%, 28.3%, and 16.9%, respectively, in newborns with Apgar score<7 at the 5th min.

The rate of low Apgar scores at 5th min varies among continents, with higher rates in developing and underdeveloped countries. In an Ethiopian study, the rate of low Apgar score at the 5th min was 13.8%^15^. In a Nigerian study, the rate of low Apgar score at the 5th min was 4.0%^18^. In our study, the rate of Apgar score<7 at the 5th min was 1.02%, but we should point out that our service is a reference for low-risk pregnancies with few maternal/fetal risk factors for low Apgar scores. In a large low-risk population sample (31.5 million deliveries), Chen and Chauban^19^ assessed the association between a low Apgar score at the 10th min and adverse perinatal outcomes. The authors observed that the rate of composite neonatal adverse outcomes was 403.5 per 1,000 live births with a low Apgar score at the 10th min (0–3). The rate of infant mortality was 201.0 per 1,000 live births among newborn infants with a low Apgar score at the 10th min. Another factor that may explain the low rate of Apgar scores at the 5th min in our study could be the small sample size.

Neonatal therapeutic hypothermia has been demonstrated to reduce death or disability in term and near-term infants with moderate–severe hypoxic–ischemic encephalopathy. The technique consists of whole-body cooling and selective head cooling. The cooling devices generally used are ice packs and phase change material for whole-body cooling and cooling caps for selective cooling^20^. Meta-analysis of 11 randomized controlled clinical trials of selective head cooling and whole-body cooling initiated within 6 h after delivery, involving 1,505 term and near-term infants (≥35 weeks gestation) with moderate to severe hypoxic–ischemic encephalopathy demonstrated the benefits of hypothermia^21^.

In our study, we used the Apgar score<7 at the 5th min as a criterion for neonatal therapeutic hypothermia. Chen et al.^22^ assessed 258 newborns (110 hypoxic–ischemic encephalopathy and 148 controls) and observed by multivariate logistic regression analysis that low birth weight (<2,500 g), amniotic fluid contamination, low Apgar score at the 1st min (≤3), and low Apgar score at the 5th min (≤7) increased the risk of hypoxic–ischemic encephalopathy. In our study, 5.3 and 25.0% of newborns who received therapeutic hypothermia had low birth weight and thick meconium in the amniotic fluid, respectively.

## CONCLUSION

The rate of Apgar score<7 at the 5th min was low, and it was not associated with any maternal/perinatal complications. Furthermore, newborns with or without therapeutic hypothermia did not differ among maternal/perinatal complications.
